# Report of Deaths in the City of Buffalo for the Month of January, 1862

**Published:** 1862-03

**Authors:** Sandford Eastman

**Affiliations:** Health Physician


*Report of Deaths in the City of Buffalo for the month of January,* 1862

Accident 1, Albuminuria 1, Bronchitis 1, Cancer 1, Cancer of Stomach 1, Cirrhosis of Liver 3, Consumption 12, Convulsions 12, Croup 6, Diarrhœa 4. Disease of the Brain 1, Disease of the Heart 3, Disease of the Liver 1, Diphtheria 3, Dropsy 2, Dropsy of the Heart 1, Epilepsy 1, Fever, Scarlet 14, Fever, Typhoid 4, Gangrene 1, Inflammation of Bowels 3, Inflammation of Brain 2, Inflammation of Brain and Meninges 4, Inflammation of Lungs 6, Inflammation of Lungs, Typhoid 1, Intemperance 2, Jaundice 1, Marasmus 3, Measles 2, Obstruction of Bowels 1, Old Age 6, Ovarian Tumor 1 Paralysis 2, Pyæmia 1, Still-born 1, Unknown 12. Total 128. Ages:—Between one and thirty days 11; between one and six months 17; between six months and one year 10; between one and three years 20; between three and five years 5; between five and ten years 7; between ten and twenty years 0; between twenty and thirty years 12; between thirty and forty years 10; between forty and fifty years 7; between fifty and sixty years 7; between sixty and seventy years 8; between seventy and eighty years 3; over eighty 5; unknown 5 Total 128.

SANDFORD EASTMAN, M. D. Health Physician,

